# Transient electronics for sustainability: Emerging technologies and future directions

**DOI:** 10.3762/bjnano.16.109

**Published:** 2025-09-04

**Authors:** Jae-Young Bae, Myung-Kyun Choi, Seung-Kyun Kang

**Affiliations:** 1 Department of Materials Science and Engineering, Seoul National University, Seoul, 08826 Republic of Korea; 2 Research Institute of Advanced Materials (RIAM), Seoul National University, Seoul, 08826 Republic of Korea; 3 Nano Systems Institute SOFT Foundry, Seoul National University, Seoul, 08826, Republic of Korea; 4 Interdisciplinary Program of Bioengineering, Seoul National University, Seoul, 08826, Republic of Korea

**Keywords:** biodegradable/bioresorbable electronics, biodegradable materials, encapsulation, fabrication strategy, transient electronics

## Abstract

Transient electronics are emerging as a promising class of devices designed to disappear after a defined operational period, addressing growing concerns over sustainability and long-term biocompatibility. Built from biodegradable materials that undergo hydrolysis or enzymatic degradation, these systems are particularly well suited for temporary implantable applications, such as neural monitors, wireless stimulators, and drug delivery vehicles, as well as environmentally benign electronics for soil or aquatic disposal. Despite their potential, key challenges remain in expanding the material set for diverse functionalities, achieving high-density integration for advanced operations, and enabling precise lifetime control through strategies such as protective encapsulation. This Perspective outlines critical opportunities and technical directions to guide the development of next-generation transient electronic systems.

## Introduction

In recent years, a growing global concern has emerged regarding the unintended consequences of material longevity on sustainability initiatives, particularly in light of the escalating crisis of plastic waste accumulation in landfills and oceans [1–3]. This shift in perspective has catalyzed interest in materials whose functional lifespans can be precisely programmed and that are capable of safely degrading under biological or environmental conditions following their use. Although this need has become more prominent in recent discourse, it has long been recognized in several application domains. For instance, biodegradable polymer-based materials have been extensively explored as environmentally benign alternatives that do not leave persistent residues [4]. Similarly, in the realm of implantable medical devices, efforts have focused on the development of implants that can naturally resorb within the body, thereby eliminating long-term retention and minimizing adverse biological responses [5]. This paradigm is particularly attractive in clinical scenarios such as neurorehabilitation [6–8], gastric recovery [9], cardiac rehabilitation [10–11], and orthopedic healing [12], where device functionality is required only for a defined recovery period. In such cases, transient electronic devices offer distinct advantages over conventional implants by providing essential sensing or therapeutic functions during the acute phase and subsequently degrading harmlessly within the body. Collectively, these trends reflect a transition in materials science from an emphasis on durability to a growing emphasis on controllable disappearance tailored to specific clinical needs. Concurrently, the rapid advancement of soft and stretchable thin-film electronic devices has created fertile ground for the convergence of flexible electronics with lifetime-controllable material systems [13]. This confluence has given rise to the emerging field of transient electronics, that is, devices engineered to function over a defined time window before undergoing complete physical degradation in situ, leaving no waste or residual materials [14]. In contrast to conventional electronics designed for permanent installation or single-use disposal, transient electronics represent a paradigm shift that redefines the relationship between a functionality and physical persistence.

**Figure 1:**
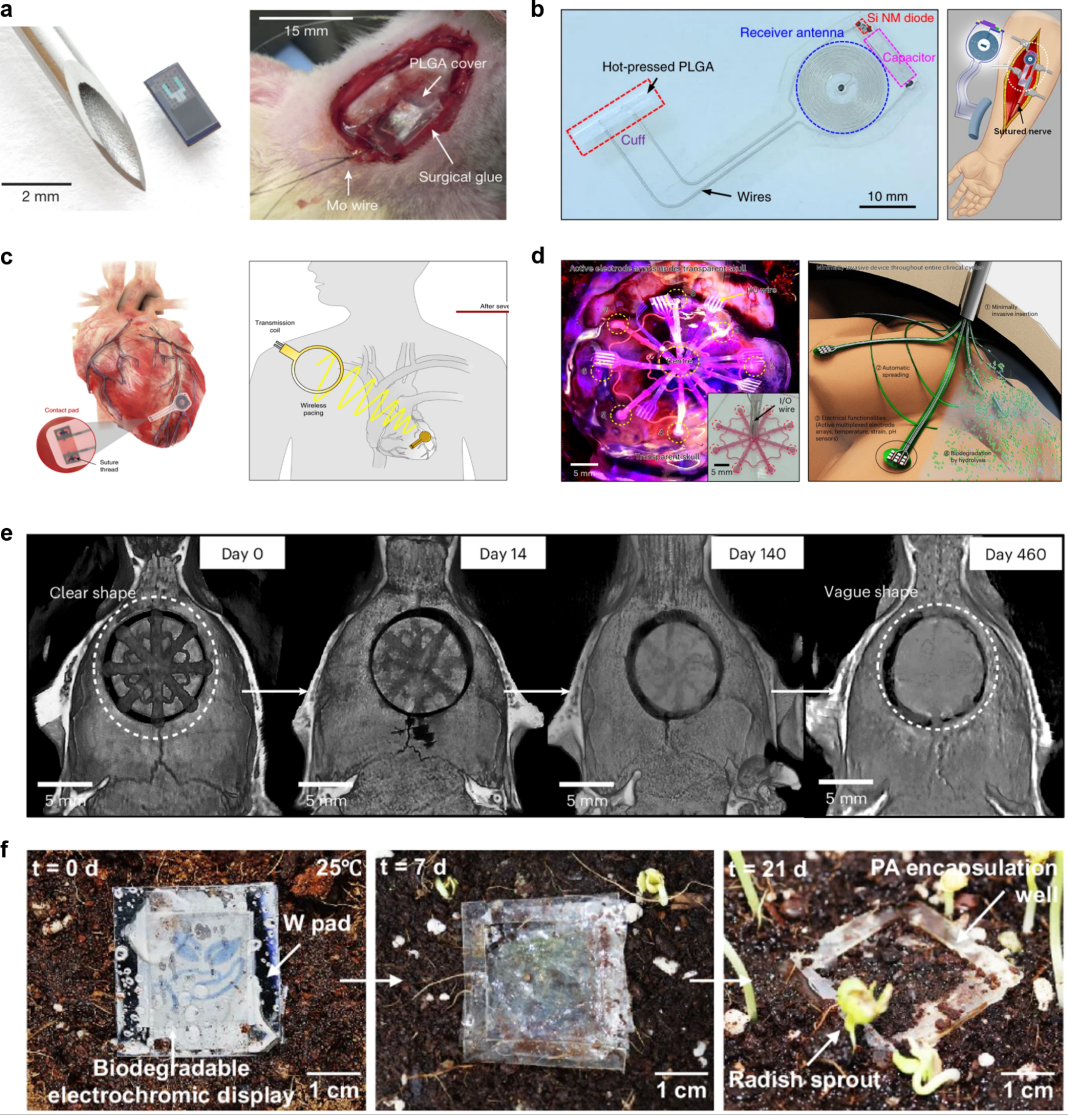
Transient electronics for implantable biomedical applications. (a) A biodegradable silicon pressure sensor (left) and its application to intracranial pressure monitoring in the brain (right). Figure 1a was adapted from [6] (S.-K. Kang et al., “Bioresorbable silicon electronic sensors for the brain”, *Nature,* vol. 530, pages 71–76, 2016, published by Springer Nature), with permission from SNCSC. This content is not subject to CC BY 4.0. (b) Biodegradable wireless stimulator (left) for functional regeneration of sciatic nerve (right). Figure 1b was adapted from [7] (J. Koo et al., “Wireless bioresorbable electronic system enables sustained nonpharmacological neuroregenerative therapy”, *Nat. Med.*, vol. 24, pages 1830–1836, 2018, published by Springer Nature), with permission from SNCSC. This content is not subject to CC BY 4.0. (c) Biodegradable cardiac pacemaker (left) with wireless and batteryless operation on heart (right). Figure 1c was adapted from [10] (Y. S. Choi et al., “Fully implantable and bioresorbable cardiac pacemakers without leads or batteries”*, Nat. Biotechnol.,* vol. 39, pages 1228–1238, 2021, published by Springer Nature), with permission from SNCSC. This content is not subject to CC BY 4.0. (d) Biodegradable and self-deployable electrode (left) for minimally invasive large-area brain interfacing (right). Figure 1d was adapted from [27] (J.-Y. Bae et al., “A biodegradable and self-deployable electronic tent electrode for brain cortex interfacing”, *Nat. Electron.,* vol. 7, pages 815–828, 2024, published by Springer Nature), with permission from SNCSC. This content is not subject to CC BY 4.0. (e) Biodegradation of transient electronics in biological domain. Figure 1e was adapted from [27] (J.-Y. Bae et al., “A biodegradable and self-deployable electronic tent electrode for brain cortex interfacing”, *Nat. Electron.*, vol. 7, pages 815–828, 2024, published by Springer Nature), with permission from SNCSC. This content is not subject to CC BY 4.0. (f) Biodegradation of transient electronics in environmental system. Figure 1f was reproduced from [32] (© 2024 S.-K. Kang et al., published by Springer Nature, distributed under the terms of the NonCommercial-NoDerivatives 4.0 International License, https://creativecommons.org/licenses/by-nc-nd/4.0/). This content is not subject to CC BY 4.0.

One of the most compelling applications of transient electronics is in minimally invasive implantable medical devices designed for postoperative care and rehabilitative medicine. During the immediate postoperative recovery period, clinical needs are typically limited to short-term monitoring or therapeutic intervention, in which case permanent implantable devices may be excessive or even undesirable. Traditional permanent implants are often associated with complications such as biofilm formation, tissue irritation, or migration within the body [15–17]. Furthermore, their removal typically necessitates additional surgical procedures, thereby increasing the risks and burden for patients [18–20]. Transient devices offer a fundamentally different approach: By biodegrading after their intended function is fulfilled, they obviate the need for retrieval and mitigate associated complications, making them ideal candidates for next-generation biomedical implants that are designed to function for a limited period, such as for temporary rehabilitation or short-term therapeutic purposes following surgery [6–8,10,14,21–30]. Clinically relevant implementations have already been demonstrated, including transient pressure and temperature sensors designed for short-term intracranial monitoring after traumatic brain injury. These devices capture delayed-onset symptoms and naturally degrade without requiring surgical retrieval [6] (Figure 1a). Examples are a wireless, fully bioresorbable electrical stimulator designed to promote nerve regeneration during the initial phase of neural injury treatment and subsequently undergo complete degradation [7] (Figure 1b), battery-free and bioresorbable pacemakers designed for on-demand cardiac rhythm management during the postoperative recovery period [10] (Figure 1c), and drug delivery vehicles developed to enable remotely triggered, programmable release of therapeutic agents, followed by complete degradation without the need for extraction [8,25]. Recent advances in minimally invasive delivery techniques have further expanded the clinical utility of such systems. Notably, devices based on biodegradable shape memory materials can be compactly delivered through narrow anatomical pathways and subsequently recover their functional form in vivo. A prominent example is a large-area transient electrocorticography array that unfolds on the brain cortex to enable neural signal monitoring following syringe-based implantation [27] (Figure 1d). The exploration of new materials and fabrication techniques has also enabled the realization of large-area transient devices. In particular, the large-area fabrication of 2D materials and van der Waals films via photonic sintering allows for the development of novel forms of transient electronic devices, thereby further broadening the scope of their potential applications [31].

The foundational principles of transient electronics rest on the deliberate selection and integration of materials, both functional and structural, that are inherently designed to disintegrate upon exposure to aqueous or enzymatically active environments. These systems are tailored to follow specific degradation kinetics, governed by hydrolysis or enzymatic cleavage depending on the chemical nature of each constituent (Figure 1e) [27]. Crucially, the utility of transient systems is not confined to the biomedical domain. Their degradation mechanisms are equally applicable in broader environmental settings where biocompatibility constraints are relaxed. In contexts such as soil, compost, freshwater, or marine environments, these devices maintain their ability to decompose through interactions with naturally occurring water and enzymes (Figure 1f) [32]. Thus, transient electronics present a compelling vision for sustainable electronics, that is, devices that fulfill their intended function and then seamlessly reintegrate into natural ecological cycles without leaving a lasting footprint [33–36].

Notwithstanding these promising prospects, several significant technical barriers need to be overcome before transient electronics can achieve widespread clinical and societal adoption [37]. This Perspective identifies three central areas that warrant focused development. First, the expansion of the available materials palette is essential to support diverse electronic functionalities. Second, advancements in high-density integration techniques are required to realize sophisticated, multifunctional devices. Third, precise lifetime control remains a critical challenge, necessitating not only improved encapsulation strategies but also dynamic, stimuli-responsive degradation mechanisms. Given the intricate nature of biological environments and the variability of therapeutic requirements, materials used in transient systems must be optimized to balance electrical performance, biodegradation kinetics, and mechanical integrity. Moreover, to transcend basic sensing and stimulation functions, and to enable capabilities such as computation, memory, and autonomous decision-making, the development of high-performance, biodegradable integrated circuits is imperative [37–39]. Ultimately, controlling the operational lifespan of such devices demands a multifaceted approach, that is, one that couples material science innovation with engineering strategies capable of responding adaptively to complex environmental cues [37,40–41].

## Perspective

### Expanding the material palette for biodegradable electronics

The elucidation and experimental validation of the biodegradation mechanism of single-crystalline silicon [14,42–44] marked a significant turning point in the development of bioresorbable electronics, offering the potential to replace conventional high-performance silicon-based devices with transient alternatives. In aqueous environments, silicon undergoes degradation via well-defined mechanisms with the rate of dissolution being highly sensitive to external conditions [42,44]. Elevated temperatures and the presence of specific ions, such as hydrogen phosphate and chloride (HPO_4_^2−^ and Cl^−^) [43] are known to accelerate the degradation process. The biodegradation rate of silicon is also influenced by its crystallographic structure. Polycrystalline silicon degrades faster than single-crystalline silicon, while amorphous silicon exhibits the highest degradation rate among them [45]. Interestingly, doping can lead to a retardation of degradation [42], resembling the etch-stop phenomenon in the potassium hydroxide process. This newfound understanding of biodegradability of electronic-grade silicon has prompted further exploration of chemically analogous materials, such as germanium [45–46], silicon–germanium alloys [45], amorphous semiconductors [45], indium–gallium–zinc oxide (IGZO) [47], and metal oxides such as zinc oxide [48], for their potential as bioresorbable semiconductors. These materials exhibit strong potential for the use in implantable medical devices. For instance, Ge nanomembranes have been employed in fully biodegradable strain and temperature sensors, demonstrating proven biocompatibility and gas-free dissolution. In addition, IGZO has been utilized to fabricate transient circuits, such as ring oscillators, entirely composed of water-soluble materials, supporting its applicability in future bioelectronic devices.

**Figure 2:**
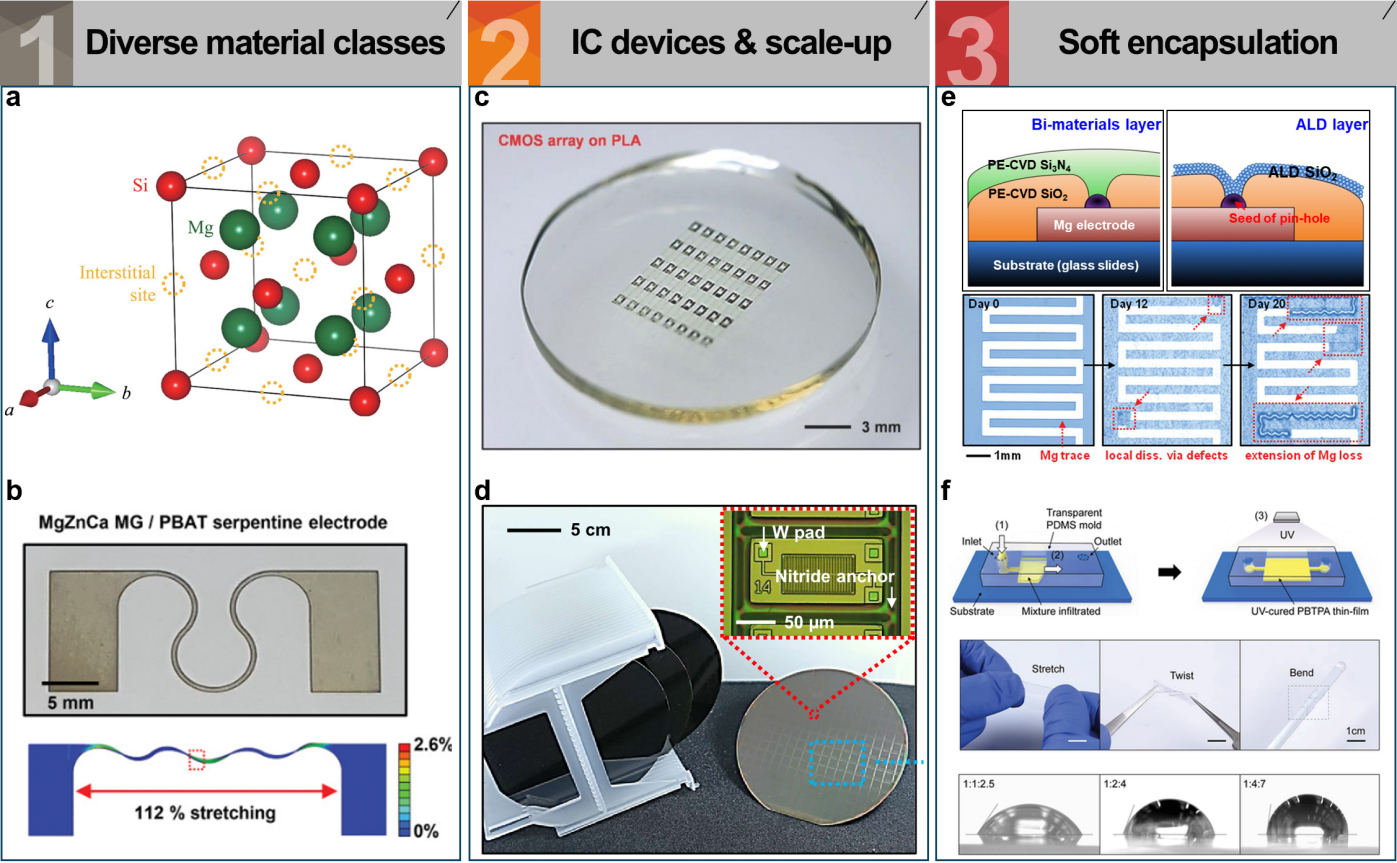
Materials, silicon-based circuit integration, and encapsulation strategies for transient electronics. (a) Lattice structure of the Mg_2_Si crystalline unit cell. Figure 2a was reproduced from [49] (© 2020 K. Hayashi et al., published by AIP Publishing), distributed under the terms of the Creative Commons Attribution 4.0 International License, https://creativecommons.org/licenses/by/4.0/. (b) Illustration of a geometrically stretchable MgZnCa electrode layered on a flexible PBAT base. Figure 2b was reproduced from [62] (© 2021 J.-Y. Bae et al., published by Wiley-VCH GmbH), distributed under the terms of the Creative Commons Attribution 4.0 International License, https://creativecommons.org/licenses/by/4.0/. (c) Biodegradable RF rectifier with passive components and antenna pads. Figure 2c was reproduced from [73], S.-W. Hwang et al., “High‐Performance Biodegradable/Transient Electronics on Biodegradable Polymers”, *Adv. Mater.,* vol. 26, pages 3905–3911, 2014, with permission from John Wiley and Sons. Copyright © 2014 WILEY-VCH Verlag GmbH & Co. KGaA, Weinheim. This content is not subject to CC BY 4.0. (d) Wafer-scale detachment of ultrathin silicon devices fabricated via foundry processes for transient electronics. Figure 2d was reproduced from [80], J.-K. Chang et al., “Materials and processing approaches for foundry-compatible transient electronics”, *Proc. Natl. Acad. Sci. U.S.A.* 114, E5522–E5529 (2017), freely available under the PNAS open access option. This content is not subject to CC BY 4.0. (e) Encapsulation strategies use bilayers to block defects, while ALD forms uniform, defect-free films; Mg degradation begins at flaws and spreads in DI water. Figure 2e was adapted from [88], S.-K. Kang et al., “Dissolution behaviors and applications of silicon oxides and nitrides in transient electronics”, *Adv. Funct. Mater.*, 24, 4427–4434 (2014), with permission from John Wiley and Sons. © 2014 WILEY-VCH Verlag GmbH & Co. KGaA, Weinheim. This content is not subject to CC BY 4.0. (f) PBTPA films, formed via capillary molding and UV curing, exhibit high flexibility and tunable wettability based on monomer ratios. Figure 2f was reproduced from [87], Y. S. Choi et al., “Biodegradable polyanhydrides as encapsulation layers for transient electronics”, *Adv. Funct. Mater.*, 30, 2000941 (2020), with permission from John Wiley and Sons. © 2020 WILEY‐VCH Verlag GmbH & Co. KGaA, Weinheim. This content is not subject to CC BY 4.0.

Nevertheless, the currently known repertoire of bioresorbable semiconductors remains narrow, both in material selection and bandgap range. Expanding the library to include materials with diverse bandgap properties remains a key challenge as it would enable wavelength-specific and electrically optimized device designs across a wide array of applications, including sensors, radio frequency (RF) devices, energy harvesters, and optoelectronic systems. For instance, low-bandgap bioresorbable semiconductors based on magnesium–silicon alloys, such as Mg_2_Si, could be promising candidates to fill this gap (Figure 2a) [49–50]. However, further exploration is needed to discover and engineer additional semiconducting materials, including a broader range of silicon- or germanium-based alloys with various metals, as well as emerging heterostructure systems, to achieve tailored electronic and optical properties suited for diverse transient device architectures.

Bioresorbable metals are also regarded as essential components for interconnects and electrodes in transient electronic systems. Traditionally studied bioresorbable metals include magnesium, zinc, and their alloy AZ31B [51–52]. Mg has been utilized as a transient conductive material in implantable pressure and temperature sensors, where it served as the interconnects and electrodes and underwent complete bioresorption in vivo [14]. Zinc has been proposed for use in bioabsorbable vascular stents, demonstrating ideal degradation behavior and mechanical integrity in animal models [51]. AZ31B, along with molybdenum and tungsten, have been investigated as a substrate and conductive layer in transient electronics due to their tunable dissolution profiles in physiological environments [52–53]. Notably, Mo and W were employed as the underlying metal foils for building metal-oxide-semiconductor field-effect transistors (MOSFETs) and PIN diodes, suggesting their utility in the development of next-generation bioresorbable electronic platforms [52]. However, the available range of bioresorbable metals remains limited. Mg and Zn exhibit rapid degradation under physiological conditions, with rates reaching approximately 1.2–12 µm/day (Mg; pH 7.4 simulated body fluid (SBF) at 37 °C) [54–57] and 3.5 µm/day (Zn; pH 7.4 phosphate-buffered saline (PBS) at 37 °C) [37,53]. In contrast, Mo and W degrade significantly more slowly with dissolution rates of 0.001 µm/day (Mo; pH 7 buffer at rt) [58] and 0.48–1.44 µm/day (W; pH 7.4 SBF at rt) [59], which is advantageous for long-term device stability but poses challenges for applications requiring complete resorption within a short timeframe. In terms of electrical conductivity, bioresorbable metals still lag conventional interconnect materials such as copper, gold, and silver, showing reductions of up to 1.96- to 3.65-fold [60].

Despite these limitations, opportunities exist to broaden the spectrum of bioresorbable metal materials. Research on Mg- and Zn-based bulk alloys, traditionally used as structural materials, has been extended to thin-film formats suitable for electronic applications. This transition enables fine-tuning of not only degradation profiles but also of electrical and mechanical performance. For instance, Mg-3Zn, which suffers from phase separation in bulk form, can be synthesized as a uniform alloy in thin films, offering improved corrosion resistance [61]. Moreover, bioresorbable amorphous metal (metallic glass) films have demonstrated large elastic strain ranges, enhancing stretchability and enabling integration with soft electronics. The Mg–Zn–Ca metallic glass, for example, has shown up to 2.46-fold improved yield strain compared to conventional Mg alloys (Figure 2b) [62]. Enhancing the diversity of metal materials is crucial to meeting the multifunctional demands of implantable bioelectronics, which require precise control over electrical stability, mechanical flexibility, and degradation timing. A versatile material portfolio enables customized device designs tailored to operation duration, implantation site, and mechanical environment, thus serving as a foundational element in the realization of high-performance bioresorbable electronic systems.

### Fabrication strategies and scalability of bioresorbable electronics

Hybrid architectures that combine the high-performance characteristics of inorganic electronic components with the mechanical flexibility of bioresorbable polymers have emerged as a representative form of transient electronics [14]. A typical example includes devices that integrate inorganic silicon nanomembranes or metal oxide semiconductors on bioresorbable polymer substrates such as poly(lactic-*co*-glycolic acid) (PLGA) or silk, enabling implantable sensors, stimulators, or power harvesters that degrade harmlessly after use. For simple sensor devices, direct deposition of inorganic materials onto bioresorbable polymer substrates using shadow masks has been employed [14,48,63–64]. For example, conductive materials such as Mg and Mo have been deposited onto bioresorbable polymer substrates in the form of resistive-type sensors or interconnects using shadow masking techniques [14,48,63–64]. In addition, semiconductor or dielectric materials such as ZnO and MgO have been patterned and deposited using shadow masking for use as channel layers or gate oxide layers in transistors [14,48,63]. Also printable electronics have been realized by blending bioresorbable fillers into polymers such as Zn (or W)-poly(ethylene oxide), Zn-polyvinylpyrrolidone, Mo-polybutanedithiol-1,3,5-triallyl-1,3,5-triazine-2,4,6(1*H*,3*H*,5*H*)-trione pentenoic anhydride (PBTPA), Mo-polybutylene adipate terephthalate (PBAT), W-beeswax, and Mo-polycaprolactone [65–71]. However, in the case of high-performance devices that require high-resolution micropatterning, the application of conventional silicon photolithography onto biodegradable substrates necessitates the use of a critical technique known as transfer printing [72–73]. One of the most sophisticated transfer printing approaches utilizes poly(methyl methacrylate) (PMMA) as a sacrificial layer and diluted polyimide (PI) as a protective layer (Figure 2c) [63,73]. In this process, PI is first coated onto PMMA, followed by photolithographic patterning of the inorganic electronic materials. The PMMA layer is then dissolved, allowing the patterned structure, protected by the thin PI layer, to be transferred onto a desired target substrate. Since the protective PI layer is extremely thin, it can be selectively removed by reactive ion etching, ultimately leaving only the biodegradable materials on the target surface. To simplify multistep fabrication processes, wafer-scale transfer methods have also been developed [74]. These involve fabricating electronic devices on a large-area substrate, such as a silicon wafer, followed by backside etching to remove the handle or box layers, thereby isolating the functional thin film for transfer onto a biodegradable substrate. This wafer-level technique offers a promising route to large-area, high-resolution, and high-throughput manufacturing of bioresorbable electronics, supporting both scalability and device integration.

Early demonstrations have successfully produced temperature [6,14,27,75–76], strain [6,14,27,77–78], and pH sensors [6,27,75,79] based on silicon and bioresorbable metals using single-step processes. However, to enable more complex functionalities, such as processing, digital communication, and memory, integrated logic devices must be developed, which inherently calls for a foundry-level scale-up. Since bioresorbable electronics can leverage existing silicon-based semiconductor processes, the substitution of conventional dielectric and metal layers with biodegradable counterparts suggests the feasibility of wafer-scale foundry integration (Figure 2d) [80]. For example, the foundry-scale fabrication of transient complementary metal-oxide-semiconductor devices (inverter, NAND and NOR) has been demonstrated using a 6-inch silicon-on-insulator (100) wafer including a silicon nanomembrane (active layer, ~250 nm), SiO2_2_ (gate oxide, inter layer dielectrics, and inter metal dielectrics of ~25, ~750, and ~650 nm, respectively), W (interconnects, ~300 nm), and Ti/TiN (via plugs, ~100 nm). The fabricated single unit of n-channel MOSFETs exhibits on/off current ratios exceeding 10^7^ and field-effect mobilities up to 680 cm^2^·V^−1^·s^−1^ [80]. This approach is considered key for realizing the high-density integration and mass production of bioresorbable systems.

Looking beyond proof-of-concept devices, future development must include circuit-level design and validation of microprocessor-grade transient electronics. Furthermore, this challenge extends beyond biodegradability and overlaps with fundamental limitations common to flexible electronics. To ensure degradability, the thickness of inorganic layers is typically limited to a few micrometers, imposing structural constraints on vertical integration schemes for wiring and packaging. These physical limitations may inherently restrict the achievable device density. To overcome these constraints, advanced nanoscale integration strategies are essential. Research needs to explore lateral integration architectures, precise microscale wiring techniques, and 2D material-based heterojunctions. These innovations will serve as a technological foundation not only for extending device functionality but also for achieving practical system-level integration in bioresorbable electronics.

### Encapsulation strategies for performance and longevity control

Transient electronic devices are designed to degrade within the body after serving their function for a defined period. However, even during use, key performance metrics, such as electrical conductivity and mechanical integrity, tend to gradually decline as the materials begin to degrade. This biodegradation is driven by the chemical nature of the materials and may occur through mechanisms such as hydrolysis, oxidation, or enzymatic reactions. The rate and pattern of performance deterioration are highly dependent on environmental factors such as pH, temperature, and ionic concentration [81]. To maintain functional performance over the intended period, a common strategy is to apply an encapsulation layer over the active components of the device. This encapsulation serves as a temporary barrier that protects the device from external environmental factors, particularly moisture. Encapsulation plays a pivotal role in the design of bioresorbable electronic devices. As these devices naturally degrade during operation, changes in material thickness and structural integrity can lead to shifts in electrical performance. Such variations may cause device malfunction or alter sensor baselines, thereby compromising accuracy. A widely adopted strategy to address this issue involves coating the outer surface of the device with bioresorbable materials that provide electrical insulation while acting as protective encapsulation [37,40,82–84]. These encapsulation layers delay the exposure of active electronic materials to moisture, enabling precise control over operational lifetime and ensuring stable performance over the desired period. However, these materials need to simultaneously block water diffusion effectively and degrade at a predetermined time, posing significant technological challenges.

Given the inherently flexible nature of thin-film bioresorbable electronics, it is critical that encapsulation layers also exhibit mechanical flexibility. As such, polymer-based solutions have been extensively explored (Figure 2f) [14,85–87]. Well-known bioresorbable polymers such as silk, PLGA, and collagen have been utilized as encapsulation materials [14]. In addition, naturally derived wax-based compounds have attracted attention due to their enhanced hydrophobicity [85]. To further improve the mechanical and crystalline properties of such natural waxes, blended composite materials (e.g., candelilla/beeswax or candelilla/PBTPA mixtures) have been investigated for their potential use as barrier coatings [86]. A 300 μm thick edge-encapsulated mixture film was found to protect a 300 nm thick Mg trace for up to three weeks in PBS (pH 7.4, 37 °C). However, organic films suffer from intrinsic porosity due to their polymer chain structure, which limits their water-blocking performance compared to inorganic counterparts.

In contrast, inorganic coatings, traditionally employed in the field of organic displays, are known for their superior water resistance due to their densely packed atomic structures. However, even these films are susceptible to defects formed during deposition, which compromise their barrier function. To overcome this, display technologies have utilized techniques such as repeated stacking of SiO*_x_*/SiN*_x_* layers via plasma-enhanced chemical vapor deposition or high-density conformal coatings using atomic layer deposition (ALD) (Figure 2e) [88]. Notably, both SiO_2_ and Si_3_N_4_ are bioresorbable materials [23,88–90], and efforts have been made to adopt these methods in transient electronics to enhance inorganic encapsulation performance. Yet, it remains difficult to completely eliminate defects within limited film thicknesses. More recently, defect-free layers formed from single-crystalline silicon or its oxide have been explored as waterproof barriers for bioresorbable devices by leveraging their thin-film formats [44,91]. The 100 nm thick thermally grown SiO_2_ layer protected the Mg pattern from PBS solution (pH 7.4, 70 °C) for 22 days. Despite these advances, conventional inorganic materials, particularly silicon-based ones, suffer from low fracture strain, limiting their mechanical compatibility with soft, flexible bioelectronic systems. Additionally, challenges in fully sealing encapsulation interfaces remain unresolved. To address these issues, hybrid organic–inorganic materials have been proposed to enhance barrier performance by artificially increasing the water diffusion pathway [92–93]. For instance, blending high-aspect-ratio inorganic flakes (e.g., SiO_2_) with a biodegradable poly(caprolactone) matrix has been shown to create tortuous diffusion paths that hinder direct water penetration, thereby improving moisture resistance and achieving a functional lifetime of over 40 days in PBS (pH 7, 37 °C) [93].

Currently, there is no single encapsulation solution that can simultaneously satisfy the demands for flexibility, water resistance, processability, and biocompatibility in bioresorbable electronics. Beyond basic moisture protection, encapsulation layers are also increasingly expected to provide resistance against mechanical shocks, electromagnetic shielding, and thermal dissipation. The development of new material systems capable of fulfilling these multifunctional requirements is essential for expanding the practical utility of bioresorbable electronics. Active degradation has emerged as a complementary strategy alongside conventional encapsulation. Unlike passive and time-dependent degradation, active degradation involves materials that remain stable under normal physiological conditions but are designed to break down in response to specific external stimuli, such as changes in pH, light exposure, or temperature [94]. Because these materials do not react under standard biodegradation conditions, they can theoretically maintain functional performance throughout the intended operational period. However, delivering such stimuli into the body without attenuation remains a major limitation, and the biocompatibility of the stimuli or triggering mechanisms may also pose challenges. Additionally, this strategy is highly material-specific, requiring the tailored design and development of functional materials for each target application, which adds another layer of complexity. Looking ahead, in addition to overcoming the limitations of active degradation materials, innovative encapsulation technologies that can actively control or even electrically manipulate fluid transport, such as through directional flow or on-demand pumping, represent a promising frontier for intelligent, next-generation transient electronic systems.

## Conclusion and Outlook

Recent advancements in bioresorbable electronics have opened new frontiers in the development of transient systems that combine high-performance functionality with biocompatible and environmentally benign dissolution. Foundational studies on the dissolution behavior of silicon and related inorganic semiconductors have enabled a paradigm shift, transforming traditional rigid electronics into soft, bioresorbable alternatives. Nonetheless, the range of available semiconductors and conductors remains limited, both in terms of material diversity and tunability of properties such as bandgap and conductivity. To achieve truly multifunctional and reliable bioresorbable systems, future research must expand the material library to encompass semiconductors with varying bandgaps, high-conductivity metals with programmable degradation rates, and mechanically compliant inorganic/organic hybrids. Equally important is the development of scalable fabrication technologies, such as wafer-scale transfer printing and foundry-compatible processes, to support the integration of logic, sensing, and memory components in a compact and disposable form factor. This task is closely linked to the identification of materials that are compatible with existing foundry processes, making the expansion of the material library a critically important challenge. Recently, as part of efforts to broaden the spectrum of usable bioresorbable materials, advanced materials such as carbon nanotubes (CNTs) [95] and two-dimensional transition metal dichalcogenides (e.g., MoS_2_) [96] are being re-examined from a new perspective, with growing interest in their potential biodegradability. The rediscovery and reassessment of such established materials will be a key research direction, especially as it aligns with the future scalability and applicability of bioresorbable and transient electronic devices. Another critical frontier lies in protective encapsulation strategies that balance mechanical flexibility, moisture resistance, and eventual degradation. While polymeric and inorganic coatings have demonstrated partial success, a unified encapsulation platform capable of addressing water barrier performance, mechanical robustness, and multifunctional protection, such as electromagnetic shielding or thermal dissipation, remains an unmet need. To overcome these challenges, active degradation strategies based on stimuli-responsive materials rather than passive, time-dependent breakdown have emerged as a promising complementary approach. As these strategies mature, they may enable the development of intelligent encapsulation layers capable of sensing and actively regulating aqueous environments, further expanding the functional scope of bioresorbable electronics. Looking ahead, the evolution of protective barriers toward intelligent interfaces that can sense, control, or even manipulate aqueous environments may further extend the applicability of bioresorbable electronics. If these technological challenges can be addressed, bioresorbable electronics are expected to evolve beyond the current paradigm of single-use, time-dependent devices into intelligent, autonomous systems capable of real-time self-assessment and self-regulated degradation. By integrating embedded logic circuits, multimodal sensors, and memory components, future systems could continuously monitor physiological conditions, initiate therapeutic interventions when necessary, and ultimately trigger complete biodegradation upon task completion.

Realizing this vision will require the development of advanced sensing platforms capable of detecting and responding to complex biological signals, alongside the integration of AI-based algorithms for real-time lifetime prediction and adaptive control. Beyond biomedical applications, such intelligent transient systems hold significant promise in broader fields, including environmentally sustainable disposable sensors, covert surveillance technologies, and secure data storage devices that leave no physical trace. Collectively, these advances may enable the emergence of a new technological paradigm, vanishing electronic ecosystems, in which electronic systems seamlessly interact with their environment, fulfill their intended functions, and then disappear without residue or retrieval. As the field matures, the convergence of materials science, device engineering, and systems integration will be essential to unlock the full potential of these transient technologies across biomedical, environmental, and security applications.

## Data Availability

Data sharing is not applicable as no new data was generated or analyzed in this study.
